# Marjolin’s Ulcer: Mesh-Related Vaginal Cutaneous Fistula With Superimposed Osteomyelitis and Neoplastic Transformation

**DOI:** 10.7759/cureus.16476

**Published:** 2021-07-19

**Authors:** Shilpa N Gajarawala, Jessica N Pelkowski, Paul D Pettit, Gregory K Lewis

**Affiliations:** 1 Medical and Surgical Gynecology, Mayo Clinic, Jacksonville, USA; 2 Orthopedic Surgery, Mayo Clinic, Jacksonville, USA

**Keywords:** chronic inflammation, malignant transformation, marjolin’s ulcer, squamous cell carcinoma, vaginocutaneous fistula, vaginal mesh complication

## Abstract

Marjolin's ulcer is a rare, often aggressive squamous cell malignancy identified in previously injured areas or those affected by chronic inflammation. It often develops in deep wounds that are slow to heal or allowed to heal by secondary intention. Few reports and small case series about Marjolin's ulcer have been published. We present a unique case with well-differentiated keratinized squamous cell carcinoma arising from a mesh-related vaginocutaneous fistula with superimposed osteomyelitis. The risk of cancerous transformation leading to Marjolin's ulcer in non-healing traumatic wounds is 8.1% and 2.6% in a fistula associated with purulent-inflammatory bone diseases. Approximately 1.7% of chronic cutaneous ulcers undergo neoplastic transformation, with a disposition to squamous cell carcinoma. Women experiencing mesh complications may require multiple procedures to address these issues and, therefore, should have them addressed in a timely manner to allow for the best patient outcome. Treatment optimization on a whole should incorporate the goals outlined by the American Urogynecologic Society and the International Urogynecological Association. These include the use of relevant evidence to help guide the management of mesh complications as well as identifying the gaps in currently available evidence, developing a treatment algorithm to be used for shared decision making, and identifying provider and healthcare facility characteristics that may optimize treatment outcomes specific to mesh complications.

## Introduction

Marjolin's ulcer is a rare, often aggressive squamous cell malignancy identified in previously injured areas or those affected by chronic inflammation. It most often develops in deep wounds that are slow to heal or allowed to heal by secondary intention [1]. Historically, Jean Nicholas Marjolin first reported malignant transformation in chronic burn scars in 1828 [1]. Rosser et al. in 1934 were the first to report seven cases of fistula that had undergone malignant transformation [2], and in a Mayo Clinic study, 4000 cases of chronic osteomyelitis were reviewed with malignant lesions found in 23% of these patients [3].

Though few reports and small case series about Marjolin's ulcer have been published, specific risk factors are still unknown about the formation of malignancy within a chronic ulcer [4]. The risk of cancerous transformation leading to Marjolin's ulcer in non-healing traumatic wounds is 8.1% and 2.6% in a fistula associated with purulent-inflammatory bone diseases [1,5]. Approximately 1.7% of chronic cutaneous ulcers undergo neoplastic transformation, with a disposition to squamous cell carcinoma (SCC) [1,5]. The average time from the time of skin injury to malignant transformation is greater than 30 years with reported range from four weeks to 75 years [6].

## Case presentation

We report a rare case of a 79-year-old diabetic female with well-differentiated keratinizing SCC arising in a vaginocutaneous fistula secondary to polypropylene mesh-related complications. Except for diabetes, medical history is negative for diseases associated with chronic inflammation such as arthritis, asthma, atherosclerosis, autoimmune diseases, and cancer.

The patient underwent an anterior Prolift synthetic mesh kit placement and trans-obturator tape (TOT) mid-urethral sling procedure in 2008 for pelvic organ prolapse and stress urinary incontinence. One-year post-procedure, she developed symptoms of pain and chronic drainage from her right inguinal and groin area with malodorous discharge from her vagina.

**Figure 1:**
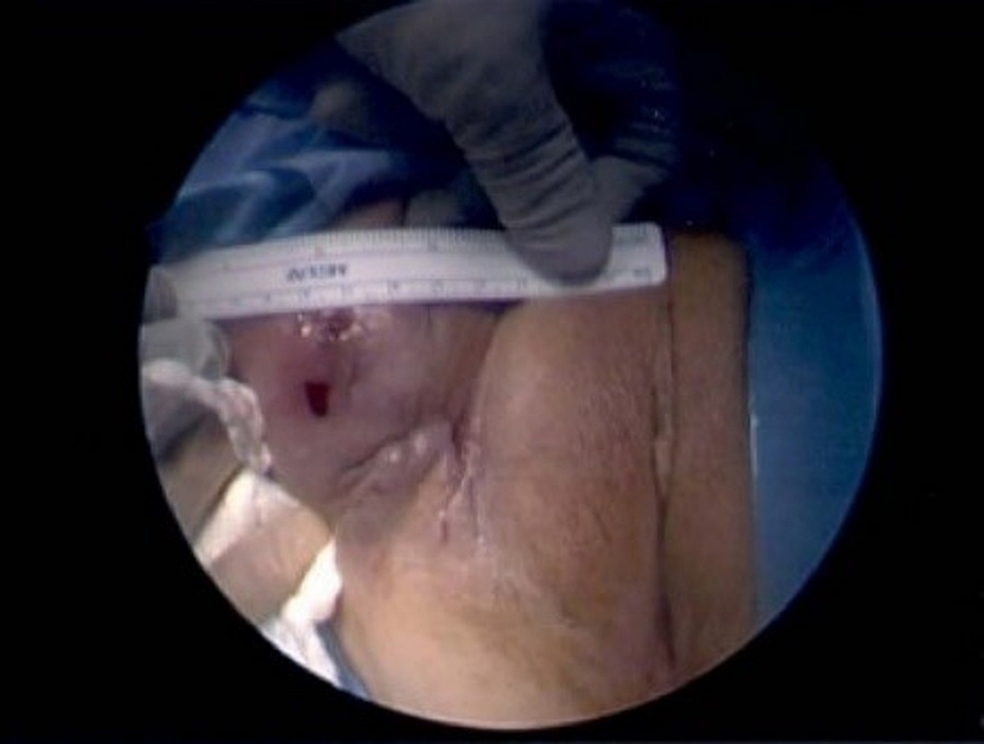
Fistulous tract, extending to right groin, measuring approximately 2 cm

Due to persistent worsening symptoms, the patient was referred to our urogynecology clinic in May of 2018, and examination showed extensive vaginal mesh erosion with a chronic draining sinus from where the mesh arms would have been placed. Her vaginal epithelium was atrophic, with a large area of visualized mesh erosion through the apex of the vagina and the anterior vagina wall. A fistula tract measuring approximately 2 cm in diameter with minimal discharge and minimal fibrinous material was noted overlying the right intertriginous region, lateral to the labia majora (Figure 1).

**Figure 2:**
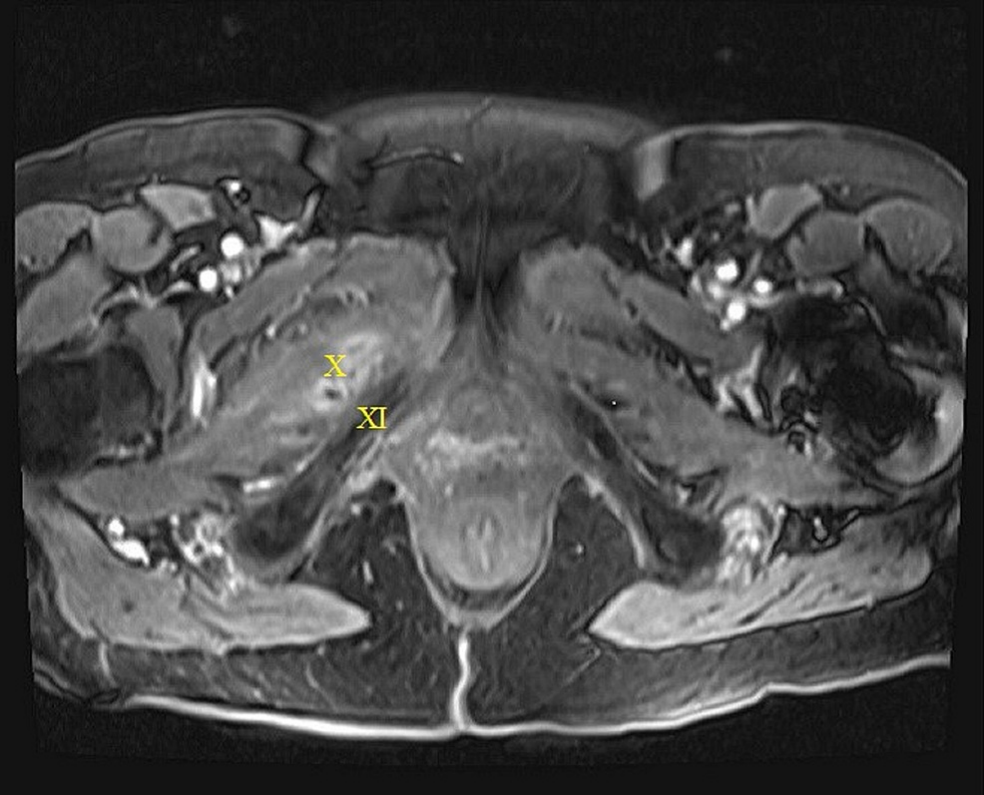
MRI taken on May 2018. Vaginocutaneous fistula tracking around the pubic ramus through the right anterior pelvic and thigh musculature and terminating at the surface of the right medial thigh lateral to the right labia X = fistula; XI = pubic ramus

MRI at that time revealed a stable fistula extending from the right vaginal cuff through the right anterior pelvic and thigh musculature and terminating at the skin lateral to the right labia (Figure 2). Subsequent cystoscopy revealed no bladder involvement. 

**Figure 3:**
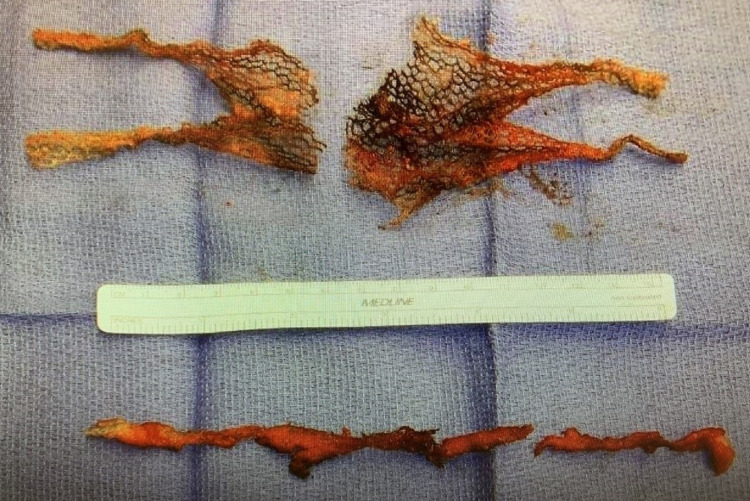
Excised mesh

The patient underwent surgery in the form of transvaginal excision of the Prolift mesh and TOT sling with dissection through the medial obturator membrane and muscle to facilitate the excision of the mesh from the left and right sides (Figure 3).

Her surgery was complicated by a left ureteral transection, which was immediately recognized and corrected by performing a left ureteroneocystostomy and placement of a left double-J stent. Cultures taken at the time of surgery grew *Escherichia coli*, *Proteus mirabilis*, and *Corynebacterium* species. The patient was initially treated with intravenous piperacillin/tazobactam followed by a complete course of 14 days of oral amoxicillin/clavulanate. 

**Figure 4:**
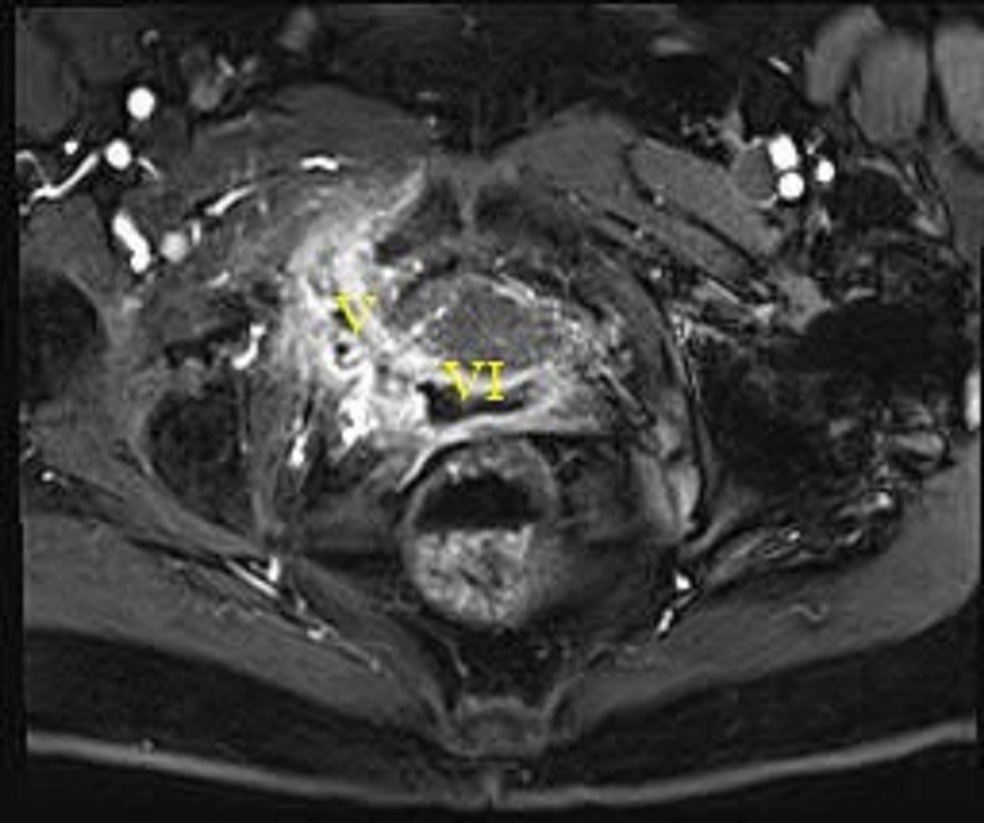
MRI. Fistula tract originating in the right vaginal corner traversing inferior to the pubic ramus V = fistula; VI = vagina

Five months post-surgery she continued to have persistent drainage from the sinus tracts in the right lateral inguinal region. MRI imaging revealed a persistent vaginocutaneous fistula with a tract length of 9 cm extending from the right vaginal cuff to the right obturator and adductor muscles to the left medial thigh. Although not obvious on imaging, it was suspected she had retained mesh (Figure 4).

She then underwent another procedure with excision of right upper inner thigh soft tissue involved in multiple chronic draining sinuses and removal of the remaining portion of trans-obturator sling, which was involved with the chronic draining sinuses. Cultures taken at the time of surgery grew *Providencia rettgeri*, *Trueperella bernadine*, *Enterococcus faecalis*, and *Bacteroides fragilis*. Inpatient parenteral antibiotics were administered for the multi-organism culture under the direction of the infectious disease specialist. She was discharged on oral amoxicillin/clavulanate. The pathology report revealed skin and soft tissue with acute and chronic inflammation reactive changes. No malignant changes were noted.

**Figure 5:**
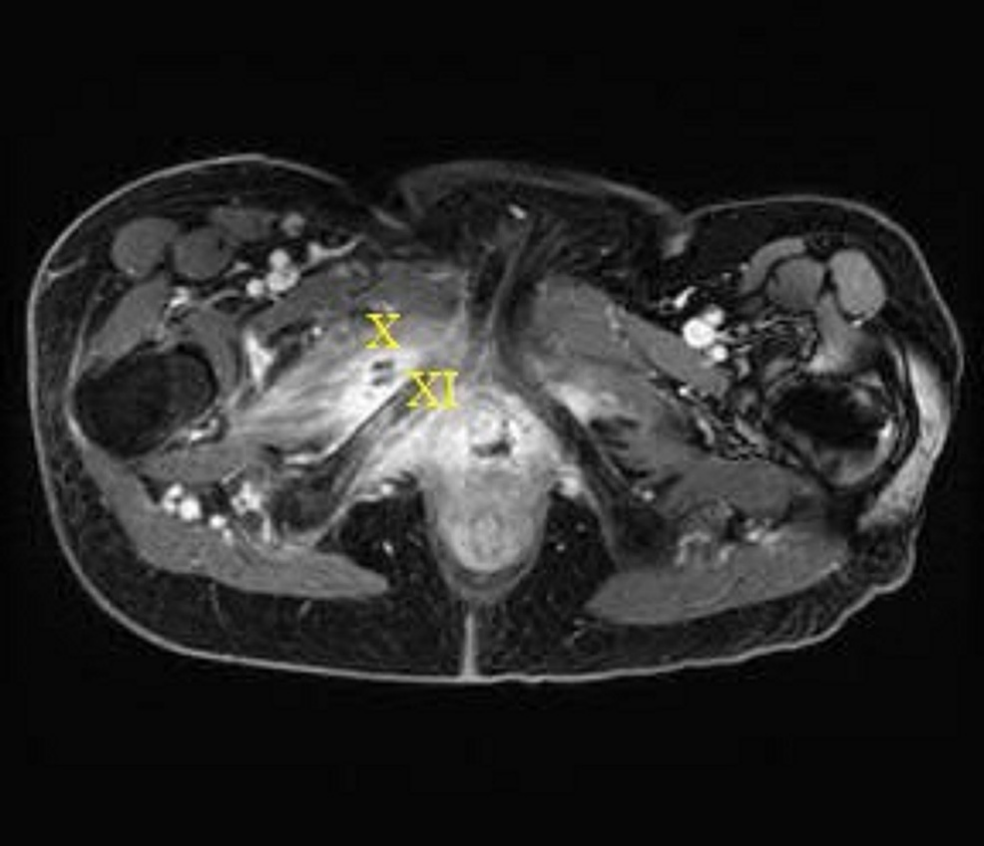
Preoperative MRI, October 2019. Persistent vaginal cutaneous fistula with non-healing right upper thigh wound/exit point X = fistula tract; XI = inflammation and edema (osteomyelitis) of the pubic ramus

The patient continued with a non-healing wound in the right upper thigh with subsequent MRI (three months post-surgery) showing a vaginal cutaneous fistula with signs of osteomyelitis of the pubic rami (Figure 5).

She then underwent another debridement and excision of the fistulous tract. Cultures grew *Corynebacterium striatum* and *Bacteroides fragilis*, and she completed six weeks of vancomycin, ceftriaxone, and Flagyl. She experienced resolution of her symptoms for about one month subsequent to this surgery. Despite chronic suppression with antibiotics as recommended by the infectious disease specialists, her postoperative course was complicated and required readmission due to complaints of increased pain and discharge from her chronic genital tract fistula. She was then evaluated by a multidisciplinary team including gynecology, orthopedics, and plastic surgeons for consideration of a more radical surgical intervention to excise the entire fistulous tract and alleviate her symptoms. She declined further surgical intervention.

**Figure 6:**
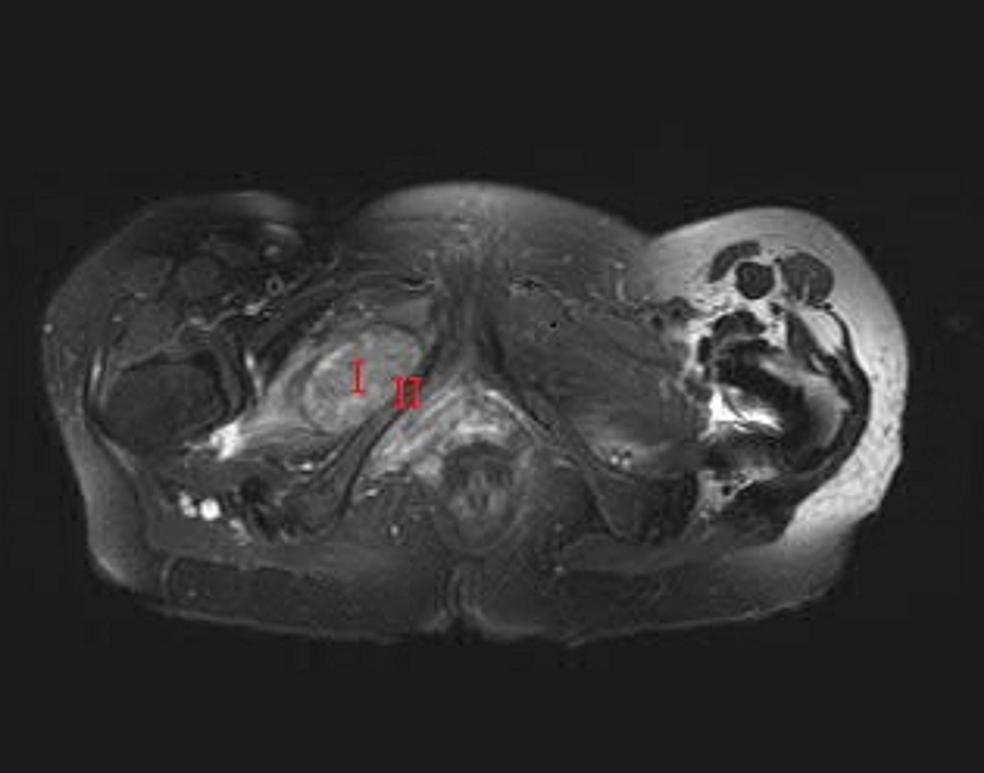
Chronic fistula with malignant transformation I = malignant  transformation in the area of previous fistula; II = pubic ramus

In October 2020 (12 years from her mesh placement and two years from her initial presentation), another MRI examination revealed continued expansion and enhancement throughout the chronic pelvic fistula concerning for squamous malignancy. Persistent edema and patchy enhancement in the right pubic ramus were noted, which reflected osteomyelitis (Figure 6). Subsequent ultrasound-guided biopsy of the right pubic mass revealed a well-differentiated keratinizing SCC.

Given these findings a multidisciplinary team approach to her management was again taken and the patient was referred to a radiation oncologist, medical oncologist, and an orthopedic surgeon. Subsequent staging in November, with a positron emission tomography (PET) scan, demonstrated slight interval growth of hypermetabolic vaginal mass extending through the right obturator foramen, hypermetabolic right obturator, external iliac, and inguinal lymph nodes indicating possible reactive, secondary chronic infection or lymph node metastasis. DNA mismatch repair deficiency was not found; therefore, pembrolizumab was not an option. Radical surgery with internal hemi-pelvectomy removing the superior and inferior pubic rami on the right side, obturator foramen, vessels, and nerves with portions of the bladder wall and vaginal wall was discussed with the patient. She again declined any surgical intervention and remained undecided about conservative management with chemoradiation. She opted to continue chronic suppression with antibiotics.

## Discussion

The use of vaginal polypropylene mesh for the surgical management of pelvic organ prolapse and incontinence in women is not without known complications [7]. These complications can occur early or late in the postoperative period and include adhesions, seromas, chronic pain, dyspareunia, erosion, or rejection of the mesh, as well as mesh-related infections [7]. In addition to the expected common postoperative complications, providers need to be vigilant about secondary complications such as Marjolin’s ulcers, reflecting malignant transformation in fistulas as was noted in this case.

The development of a keratinized, well-differentiated SCC arising from a vaginocutaneous fistula is extremely rare [2]. Although there is no clear consensus on malignant transformation, the most widely accepted theory involves chronic inflammation with immune system dysregulation [8]. Immune system inflammatory mediators and cytokines are thought to regulate various proteins' genic expression, including p53 [5]. There is evidence that a shift in bacterial flora from gram-positive to gram-negative flora can induce carcinomatous transformation. Malignant transformation can occur through DNA damage and the simultaneous promotion of tissue destruction and repair related to infectious agents such as gram-negative endotoxins, which trigger inflammation, via recruitment of cytokines and growth factors to the infected site [9]. SCC is distinguished by an intraepidermal proliferation of atypical keratinocytes [10]. Our patient did have significant bacterial organisms associated with her chronic fistula. SCC is distinguished by an intraepidermal proliferation of atypical keratinocytes [10].

As a result of Marjolin’s ulcer's greater aggressiveness, compared to other skin neoplasms, a timely diagnosis and a well-designed treatment plan are mandatory to optimize the patient's medical care and outcomes [11]. Providers should maintain a high index of suspicion in patients who report increased pain, blood, or purulent drainage from the sinus, progressive osteolysis and erosion, and an enlarging mass in the wound area [12,13]. In addition to a thorough history and physical examination, primary and secondary imaging modalities will facilitate diagnosis. MRI can help differentiate SCC from other soft tissue cancers [14]. Whole-body PET-CT can be used to evaluate for metastatic disease. These imaging modalities were judiciously used in our index patient and did guide us to her eventual SCC diagnosis.

Due to the paucity of clinical information surrounding this malignancy, no standards have been established for diagnosis and treatment [6]. Common treatment recommendations include complete local excision and en block excision of regional lymph nodes. If this is not possible, it is recommended to obtain surgical margins, with amputation of large neurovascular structures adjacent to the advanced lesion. It is prudent to obtain a complete surgical excision with a range of at least 2 cm, including any thick scar tissue in the ulcer margins [6]. Additional treatment options for patients with unfavorable prognostic factors or remote metastases include neoadjuvant or adjuvant therapy, such as radiation therapy and/or chemotherapy [15,16]. Local radiation can be considered for supplementary therapy or primary therapy based on tumor size or location if complete resection is not possible or if the patient declines surgery as was the case with our patient. An aggressive multimodal approach combining preoperative chemoradiation and adjuvant chemotherapy may achieve favorable effectiveness, response rates, and outcomes [15,17,18,19]. Neoadjuvant therapies may be options to decrease the tumor's size before the surgery. There exists concern regarding cutaneous radionecrosis negatively affecting tissue repair and outcomes [15,18].

Treatment optimization should include the following goals: use of relevant evidence to help guide the FPMRS subspecialist caring for patients with mesh complications, a treatment algorithm that can be used for shared decision making, identify and prioritize gaps in evidence related to specific mesh complications and treatment options, and identify provider and health facility characteristics that may optimize treatment outcomes specific to mesh complications [20].

## Conclusions

Our experience with this patient validates the importance of timely diagnosis and treatment. Providers, who treat chronic wounds, should be mindful of the risk of malignant transformation. In addition, lymphatic spread and organ metastasis consequences should not be overlooked.

Due to the lack of available data and definitive therapeutic guidelines, this report aims to present our experience based on the outcomes after consideration of multimodal therapy (chronic suppressive antibiotic therapy, surgery, chemotherapy, and radiation therapy) for management of malignant transformation in a patient with a chronic non-healing vaginocutaneous fistula due to mesh complications.

Treatment optimization for mesh complications should include the following goals: use of relevant evidence to help guide the FPMRS subspecialist caring for patients with mesh complications, a treatment algorithm that can be used for shared decision making, identify and prioritize gaps in evidence related to specific mesh complications and treatment options, and identify provider and health facility characteristics that may optimize treatment outcomes specific to mesh complications.
